# Recent Progress Using De Novo Design to Study Protein Structure, Design and Binding Interactions

**DOI:** 10.3390/life11030225

**Published:** 2021-03-10

**Authors:** Juan Ferrando, Lee A. Solomon

**Affiliations:** 1 Department of Biology, George Mason University, 4400 University Dr, Fairfax, VA 22030, USA; jferrand@gmu.edu; 2 Department of Chemistry and Biochemistry, George Mason University, 10920 George Mason Circle, Manassas, VA 20110, USA

**Keywords:** de novo protein design, binding, protein-protein interactions

## Abstract

De novo protein design is a powerful methodology used to study natural functions in an artificial-protein context. Since its inception, it has been used to reproduce a plethora of reactions and uncover biophysical principles that are often difficult to extract from direct studies of natural proteins. Natural proteins are capable of assuming a variety of different structures and subsequently binding ligands at impressively high levels of both specificity and affinity. Here, we will review recent examples of de novo design studies on binding reactions for small molecules, nucleic acids, and the formation of protein-protein interactions. We will then discuss some new structural advances in the field. Finally, we will discuss some advancements in computational modeling and design approaches and provide an overview of some modern algorithmic tools being used to design these proteins.

## 1. Introduction

Binding is one of the most important and fundamental biological processes, underpinning everything from drug interactions to genetic regulation and cytoskeletal formation [1,2,3]. Although no two binding interactions are exactly alike, there are common biophysical driving forces that we can extract to better understand how natural systems operate. However, direct studies of proteins are difficult due to the inherent complexity brought on by millions of years of evolution [4,5]. Natural selection has given rise to all proteins necessary for life to exist but has brought with it a host of functions and interactions we still do not understand. The pH, temperature, surface and molecular crowding of the environment in which the protein is synthesized impacts its function greatly, adding further complications [6].

**Figure 1:**
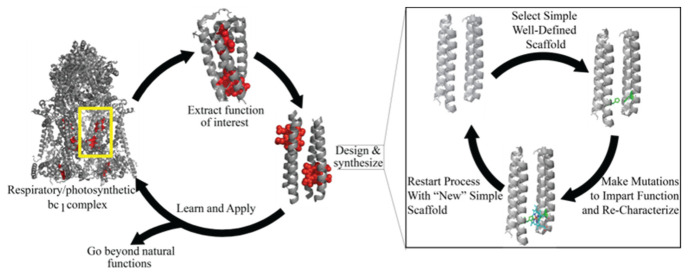
Process flow of De Novo protein design. Starting with a specific protein, the function of interest is isolated. A protein is modified from basic chemical principles so that it reproduces that function. In so doing, the designers can learn and rigorously test the underlying biophysical principles. The design and synthesis process itself begins with a simple scaffold. Mutations are made to impart function and the whole protein is recharacterized. If function is not achieved, or achieved to a sufficient level, the process restarts, however the modified protein is the new scaffold. In this iterative process complexity is kept to a minimum. An important aspect of de novo design is that proteins can be used in abiological contexts. This allows the expansion of natural functions into areas of synthetic chemistry making the de novo proteins a versatile tool capable of addressing many issues.

Protein design avoids many of these pitfalls by starting with a well-understood scaffold that has either a modest or no direct relation to natural sequences. Changes are introduced based on chemical principles rather than mimicry, which removes a lot of the potential for mutational side effects that would otherwise bias the results away from the intended chemistry being studied. A simplified approach to protein design is as follows (Figure 1) [7]: First a target function is selected and a nonfunctional scaffold protein is chosen alongside that. The scaffold is mutated until the function of interest is reproduced. The final mutations are analyzed to see what biophysical and biochemical parameters are changed or incorporated that allow for the initial target function to be achieved.

There are different approaches to protein design, which vary mainly by how the mutations are introduced. These include directed evolution, redesign of natural proteins and de novo design. In this review we are primarily focusing on de novo protein design, which does not rely on naturally occurring proteins or mimicking sequences from nature, but rather starts from a totally abiological scaffold and introduces mutations based on the chemical principles needed for function and uses either knowledge-based or computational modeling to predict how the protein will fold and reproduce the function of interest. Since the first demonstration of this approach, simple peptides that folded into a four-α-helix bundle [8,9], it has seen many successful functional reproductions including gaseous ligand binding and organic chemical catalysis [10,11,12,13].

Though we are mainly focusing on de novo design we will touch on examples of directed evolution [14]. Directed evolution takes a brute force approach to generating functions, generating hundreds to thousands of mutational variants and searching for the most successful among them [15]. This library design can be performed many times until a specified trait (e.g., some desired rate or affinity for a small molecule) is reached. This approach has been immensely successful reproducing certain biological functions and producing many abiological functions in protein scaffolds [16,17,18], culminating in the 2018 Nobel Prize for chemistry.

In this review we are looking at the recent progress in protein design efforts towards understanding biological binding, an area very important to fields such as drug design and enzymology. Our primary focus is on papers that have been published in the past three years (2017–2020). In addition to the work of small molecules we will also be looking into DNA binding and the design of protein-protein interfaces. For more in depth perspectives on de novo protein design that both describe the methodology and provide more history and context to the field we suggest the following reviews and perspectives [5,19,20,21].

## 2. Small Molecule Binding Proteins

Small molecules are seen throughout biology, from metabolites and cofactors to drugs and therapeutics and protein design has long been interested in understanding these interactions. Here, we will go through some of the recent progress in the field describing binding interactions starting with metal ions, that can be held in place via coordination bonds, and progressing to organic molecules, that must be held in place through non-polar interactions. We will discuss recent work in the field related not just to the binding itself but also to the methods and techniques used to develop the proteins.

### 2.1. Metal Binding Proteins and Related Functions

Metal ions represent the smallest possible ligand that can impart a function. Thorough characterization of natural and model systems have provided many concepts that have been used by protein designers to incorporate transition metals into de novo builds to advance function and obtain new insights into natural proteins. We begin our review on these seemingly simple ligands and the de novo proteins that can bind and use them for catalytic functions.

Early work in this field used common metal binding sites grafted into de novo protein interiors [22,23], but Kaplan and DeGrado generated a Due Ferri (DF) protein inside a four-helix bundle from first principles [24]. The bound Fe ions were able to catalyze the two-electron oxidation of 4-aminophenol to its monoamine form in an artificial context. This work was impactful and formed the basis for many other metal binding proteins since.

Recent work has expanded this work to a new series of metals. Paredes et al. formed a protein-titanium complex with the Due Ferri single-chained scaffold (DFsc) [25]. They were stabilized by two equivalents of titanium IV within a protein scaffold and could hydrolytically cleave DNA making it the first soluble titanium protein complex. This reaction utilizes one of the most abundant transition metals in the earth’s crust and will greatly reduce the cost of complex chemistries by decreasing the need for rare earth metals.

Recently, dinuclear Mn clusters to were incorporated into the DF scaffold to develop a model system to study electrochemical Mn reactions [26]. Olsen, Allen and others took the four-α-helix DFsc protein and modified the Fe-binding site to generate five distinct proteins that ligate Mn at different positions in the bundle architecture, all of which were confirmed by electron paramagnetic resonance (EPR) spectroscopy. These proteins reproduced the functions of Mn Catalase and took part in electron transfer similar to the Mn cluster of PSII. This work establishes a new model system for Mn based catalysis and will shed light on water splitting reactions seen in plants and help exploit those reactions for other technologies in the future.

Zhang and Lombardi et al. have taken another step closer toward reproducing a full Mn cluster seen in PSII by designing a protein capable of binding a tetra-zinc cluster [27,28]. Their design uses the four Zn atoms to form anchor points for four separate helices resulting in a homotetrameric assembly. This work avoids Cys and His ligation of metals in favor of Asp residues to hold the metal in place, an important design challenge. They were also able to design in a network of hydrogen bonds to stabilize the Zn-cluster in this non-natural configuration. This work is important as it could lead to reproductions of the Mn cluster from the oxygen evolving complex of photosystem II in plants.

In 2020 Pirro et al. designed a two-domain de novo protein called DFP1 (Due Ferri Porphyrin) which is capable of oxidizing phenol after binding to a synthetic Zn-porphyrin [29]. One of the domains is from the Due Ferri family and the other is based on PS1 (Porphyrin-binding sequence). When synthetic Zn-porphyrin binds to the PS1 domain it causes changes in structure which influence the Due Ferri domain’s catalytic rate. This design is significant because it is capable of allosteric communication and uses that communication to drive function. The authors state that their design strategy differs from prior known strategies like through domain insertion and fusing two protein domains in which one or two linkers resulted in the domains fusing end-to-end.

Expanding the work of the DF proteins, Mancini, Nanda, and others developed a protein that bound a larger cubane metallic cluster [30]. They computationally redesigned a 4-helix bundle protein to provide ligations to hold a Fe_4_S_4_ cluster inside the core of the bundle. The initial scaffold was selected based on its high tolerance for mutations, so this protein has a lot of potential to reproduce a wide array of redox functions of many natural proteins. This work will be important to expanding the functional library of de novo proteins and provide the ability to finely tune an important functional cofactor with high confidence in the stability of the scaffold.

Selvan and others used rational design to change the function of a different protein scaffold, Cu storage protein (Csp1), into an artificial metalloenzyme (ArM) capable of hydrogenase activity [31]. Their approach starts with identifying key residues to covert Cu binding sites to Ni and Fe. However, the authors then validated their work using the NAMD computational modeling program and QM/MM simulations. They confirmed reactivity through in vitro experimentation, showing faradaic efficiency in Hydrogen reduction with a high turnover number and expected pH dependent mechanism. The authors made minor modifications to an established 4-heilx bundle scaffold to take advantage of the predefined binding site and engineer new Ni and Fe binding properties. Their work shows a powerful use of rational design to alter the function of a protein through application of chemical principles.

Mutter and coworkers designed a protein that can bind an FeS cluster, a common redox cofactors from biology, and link this metal cluster to electron transfer reactions with other proteins in vivo [32]. They designed these proteins through phylogenetic analyses that produced a consensus sequence which binds two FeS clusters in symmetric sites. Importantly, the authors characterize the effect of outer shell amino acid interactions, and how that can change structural properties, but not the function. This work extends the metal-binding protein work forward by linking design efforts to evolutionary steps and demonstrating activity in vivo.

Protein function is often tied to its ability to change conformations based on the environment. Boyken et al. described a strategy to design pH responsive proteins capable of conformational changes [33]. This strategy involved pre-organizing helical bundles with histidine-based hydrogen-bond networks that would become protonated at low pH. The disruption of the hydrogen-bond network led to conformational changes which could easily be tuned by the modularity of the design. This work brings new levels of control to de novo designed systems, imparting a novel way to change the conformation and regulate function.

### 2.2. Porphyrin Cofactors

Metal-centered cofactors were amongst the earliest molecules bound to de novo proteins owing to their ease of detection and well-described principles for binding. Prevalent among these studies has been Heme B (Fe protoporphyrin IX), which was used to describe a myriad of biological functions in addition to providing the foundation for other small molecule binding designs in the future [13,34,35]. One of the most common designed protein-fold for heme binding has been the four-helix bundle, but recently Nagarajan and coworkers designed a new fold that can accommodate this functionally diverse cofactor with a K_D_ of 730 µM [36]. They designed a beta-hairpin turn with tryptophan residues to sandwich the heme in the binding pocket, and His residues to directly ligate the heme into place. This protein was verified to bind heme using a combination of NMR and UV-Vis absorbance data. This work breaks with decades of heme-binding design by introducing a beta-sheet pincer motif to ligate the cofactor, greatly opening up new functional possibilities in the future.

Zambrano et al. provided evidence that a de novo designed miniaturized heme-enzyme, named Mimochrome VI (FeMC6), can be used in hydrogen peroxide assays and even overcame prior natural catalysts in the standard luminol oxidation test [37]. Currently the standard method of detecting hydrogen peroxide involves introducing the chemiluminescence reagent luminol (which emits light at 425 nm when oxidized) along a hydroperoxide catalyst named horseradish peroxidase (HRP). The researchers began with a Mimochrome (MC) scaffold which consists of “two small peptide chains covalently linked to deuteroporphyrin in a helix-heme-helix sandwich”. The designed catalyst showed linearity within two ranges when plotted against luminescence, 10.0 µM–120 µM H_2_O_2_ and 120 µM–500 µM H_2_O_2_. The detection limit was 4.6 µM H_2_O_2_ and quantitation limits for the two datasets were 15.5 µM and 186 µM H_2_O_2_. When using HRP as a catalyst the plot shows a nonlinear response in the presaturation phase and was only able to detect hydrogen peroxide in the mM concentration range. The authors stated this assay could be used to monitor the quality of water during and after advanced oxidation processes. Advanced oxidation processes are a viable option for water reclamation for potable reuse.

Non-Fe porphyrins have also been studied with protein design. Polizzi et al. used computational methods to design a 4-helix bundle that tightly bound a tetra-CF_3_ Zn porphyrin with a 45 nM affinity that remained stably folded at >120 °C and could last for over one year [38]. This is a major step forward in this field as the porphyrin did not have any strong possible bonding interactions to anchor it into the protein, the binding had to be maintained through strictly nonpolar interactions. Impressively the authors were still able to get a high affinity and predict the final solved structure with sub-Å accuracy. Their work brings will help improve computational methods and remove the need for library-based optimization for small molecule binding.

Kodali et al., in their attempts to replicate the light-harvesting abilities of natural systems designed four-helix bundles that bound light-harvesting Zn-porphyrins with nM affinity [39]. Binding the cofactor shifted the absorbance spectra and increased the structural stability of the protein. The proteins can be designed to accommodate two distinct light-harvesting cofactors as well, and energy transfer between them was observed. Importantly, the authors were able to demonstrate a need for amphipathic character in the cofactor. Tetracarboxyphenyl porphyrin and Tetraphenyl porphyrins both bound 5-times weaker than porphyrins with defined hydrophobic and hydrophilic faces. This work will be useful going forward by reproducing a key function of biological energy generation, which will help groups exploit it to combat climate change.

### 2.3. Hydrophobic Molecules

The field of hydrophobic molecules has seen the most advancement as of late as it is arguably the most challenging due to a lack of easy to form ligation bonds clear signals to detect successful binding. Recent progress in this area has led to the creation of a new functions integrating the small molecule ligands.

**Figure 2:**
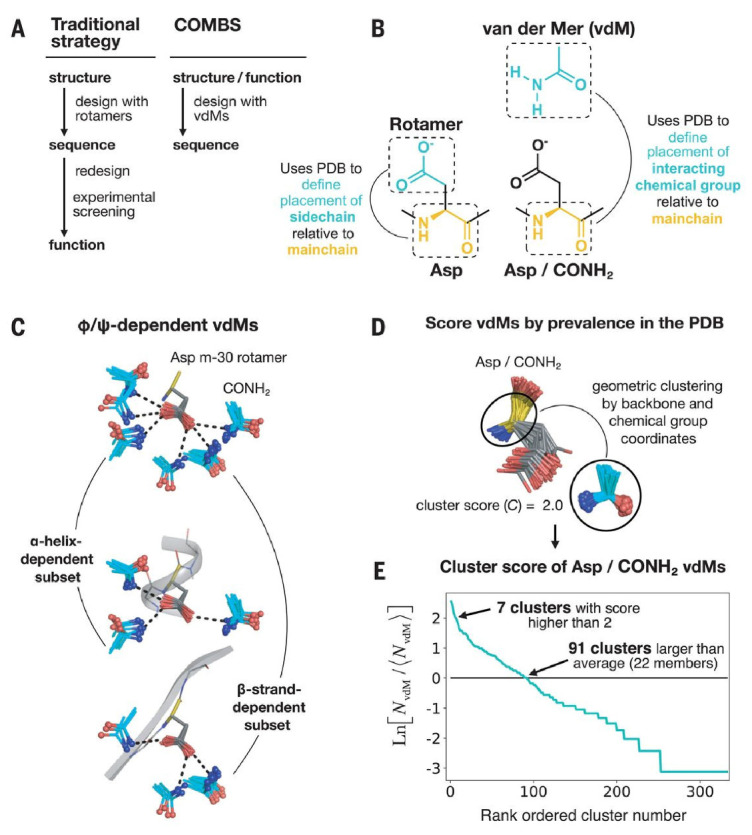
A depiction of the workflow of the new van der Mer unit. (**A**) The classical workflow of traditional protein design versus the COMBS methodology described in the paper [40]. (**B**) Definition of the van der Mer unit, accounting for the distance between the backbone Cα and the small molecule chemical group. (**C**) Next step of van der Mer modeling highlighting the rotamer and ϕ and ψ angle dependence. (**D**,**E**) Ranking of the prevalence of the chemical group-protein pair in the PDB and cluster score. The ideal amino acid side chain based on this analysis, and considering other possible interactions in the scaffold, is then selected for analysis. This work was reprinted with permission from: A defined structural unit enables de novo design of small-molecule-binding proteins. Polizzi, N.F., DeGrado, W.F. *Science* 2020, 369, 1227–1233. Copyright (2020) AAAS.

Recently, the Degrado lab has developed a new metric to assist design: the van der Mer (a combination of van der Waals and rotamer), which models the phi and psi angles of the amino acid backbone Cα, and its distance to a chemical groups, rather than atomistically model specific side chain interactions with chemical groups on the target molecule [40]. Their design strategy then takes a statistical approach based on solved crystal structures to determine the best functional group that can accommodate the chemical group (Figure 2). They used this technique to develop an apixaban binding protein achieving a µM affinity and an impressive overlap of their predicted and solved structures. Going forward, this approach promises to simplify the design of a large number of small molecule binding proteins by expanding the potential sidechain functional groups that can be incorporated.

The Baker lab has also been influential in designing proteins that can model in very specific hydrogen bonds. Their protein design approach uses Rosetta to design a binding site that locks the ligand into a very specific orientation within the core of the protein. Park et al. used this methodology to create a homo-trimeric protein capable of binding to the small molecule drug amantadine [41]. In 2019 the group was able to design an amantadine binding site where each protein monomer within the homo-trimeric protein interacts with the small molecule amantadine identically. 19 residue changes were made to 2L6HC3_13. Residue changes at Ser-71 (meant to aid in hydrogen bonding with the amino group of amantadine) along with changes at Ile-64, Leu-67, and Ala-68 (which provide a shape complimentary binding pocket) were key in the design. Amantadine binding protein (ABP) was expressed in *E. coli* where through further testing proved its ability to bind to amantadine. Although the group was not able to create an inducible trimer, they were able to identify two major bottle necks for designing such a system which further research can build upon.

In 2018 Dou et al. successfully created the first de novo designed β-Barrel capable of binding to a small molecule and fluorescing [42]. The two proteins were called mFAP1 and mFAP2. There were two advancements the group made during this study. First was establishing principles for designing a stable β-barrel. Second was designing a binding cavity with sidechains that would bind with the small fluorescent molecule DFHBI and hold it in its planar Z conformation. The authors state their approach to binding differs from prior methods “which has relied on repurposing naturally occurring scaffolds”. The designs were then expressed in *E. coli*, mammalian, and yeast cells where they were found to fluoresce in vivo.

A significant challenge in the field is discrimination between molecules with similar chemical character, but the need to distinguish one pharmacophore or odorant compound is important in nature and to many chemical industries. Thomas and coworkers have been able to address these issues using a series of designed alpha helical bundle proteins [43]. They increased the internal pocket size by creating bundles with five six or seven helices and were able to show discrimination between similarly lipophilic molecules. All three bundles sizes were able to bind palmitic acid, but only the seven-helix bundle was able to bind beta-carotene. This type of discrimination provides a sized-based set of design rules for proteins that target a molecule with many pharmacophores or naturally occurring analogues. The authors show how to tune the protein core to match a specific molecule efficiently despite a minimal number of chemical “hooks” for the protein to grab.

A common theme in many of the protein design papers discussed here is the need for a large pocket that can accommodate many substrates, and to which selectivity can be later programmed in. Certain groups approach this challenge by expanding the number of helices to increase the pocket size, but Caldwell, Haydon, and colleagues chose a different design strategy, they carved out space utilizing a previously designed de novo TIM barrel formed from two dimers; they removed sufficient bulk from buried residues, so a channel was formed [44]. Inside this channel they placed Glu residues to ligate a Tb atom, which can be used for a variety of chemical reactions. This work is a notable step in the development of artificial enzymes; the TIM barrel architecture provides a large sample space to switch out amino acids to take part in different chemical reactions. The addition of a pocket that can incorporate metals, or other cofactors, further expands this functional potential. Furthermore, this modified TIM barrel can accommodate a new class of small molecule substrates that naturally occurring examples could not.

As an example of how variable libraries can be used to develop cavities and small molecule binding sites, Karas and Hecht developed libraries based on the de novo designed 4-helix bundle protein S-824 [45]. The library created by the authors consists of 1.7 × 10^6^ unique sequences. The group characterized variant proteins from the library and demonstrated that many of the variants can withstand different amino acids in its cavity. The protein variants also contained buried polar residues that could be used for catalysis. One of these variants, Syn-F4 was proven to be a catalyst after expressing the sequence in *E. coli*. Syn-F4 was able to “rescue” another life-sustaining gene from deletion. The authors believe their library can be used to screen and select for novel proteins with different functions not evolved by nature.

Binding of small molecules can also be used to drive further functions. Kang et al. used binding sites to drive chemically induced dimerization [46]. The authors used phage display to generate a pair of proteins that only dimerize in the presence of cannabidiol, a medicinal small molecule. They used this attribute to run ELISA assays detecting it in solution, something that cannot be done solely with antibodies. Their selectivity for cannabidiol over the closely related molecule tetrahydrocannabinol was impressive as the dimerization was not seen at significant levels despite minimal structural differences. This work is notable as it can be applied to a variety of small molecules and lead to new biomolecule sensing technologies that do not rely on mass spectrometry techniques, but rather clear colorimetric assays.

In 2020 Vivek Prakash et al. designed a heterochiral de novo minimal fluorescent protein that can be selectively excited at 342 nm [47]. β-(1-azulenyl)-L-alanine, an unnatural amino acid was inserted into the hydrophobic core of a heterotactic protein scaffold, which allowed them to use automated design tools like automated repetitive simulated annealing molecular dynamics and IDeAS when designing their protein. The authors also explore different chain stereochemistries within their design. Here, we see what is possible when artificial amino acids are combined with de novo design to create a desired effect.

A primary motivation behind binding to a small molecule is to incorporate them into chemical catalysis. The majority of the papers discussed above use small molecules that resemble drugs or metabolic intermediates or other cofactors in other biochemical reactions. Below are a small number of recent examples that highlight some interesting chemical catalysis reactions.

Recently de novo design has led to the creation of C45, a tetra-α-helical c-type heme containing peroxidase protein. In 2019 Stenner et al. proved C45 can catalyze the stereoselective transfer of carbenes to olefins, heterocycles, aldehydes, and amines. The group also proved that C45 can catalyze ring expansion of aromatic heterocycles [48]. The next year Stenner et al. proved that C45 can also be used to catalyze the carbene to N-H insertion of aminophenols chemoselectively in the presence of a hydroxyl group [49]. The authors state that to their best knowledge this is “the first demonstration of an enzymatic chemoselective N-H insertion in the presence of a unprotected hydroxyl group” making their findings significant. These findings demonstrate that de novo designed proteins can be useful in catalyzing new reactions.

## 3. Transmembrane Proteins

A substantial percentage of naturally occurring proteins are situated in membranes, used for a wide variety of signaling, transport, and other functions [50,51,52]. Protein design is currently being applied to uncover these interesting features as well as many of them incorporate binding interactions.

Recently, Mravic et al. published work showing the design of a highly stable membrane protein and how, through their design process, they uncovered fundamental engineering principles to construct others [53]. Their work began by looking at a naturally occurring protein PLN, which has a 5-heilx membrane spanning domain. The authors extracted packing interactions between Ile residues at the helical interfaces and applied them to contemporary designs. The de novo proteins developed using this approach were incredibly stable due almost entirely to those interfacial packing interactions. This work provides an excellent guide toward developing new membrane spanning folds that are robust enough for further functional modifications. Importantly, this work also clarifies the role of hydrophobic and interfacial packing on the stability of membrane proteins. Points that have been long debated.

Curnow, Anderson and colleagues have also designed a transmembrane protein [54]. They used a simple sequence space to develop a protein that can insert into the membrane of both prokaryotic and eukaryotic cells. Interestingly they show that a simple set of small amino acids Gly, Ala, and Ser, when placed in key positions of the α-helix, improved the packing of the helices in the membrane. These proteins were designed to bind heme B, and carry out simple peroxidase functions with it. Interestingly, the proteins as expressed were able to scavenge free Zinc protoporphyrin IX, suggesting a primordial function of membrane proteins. This work describes a simplified membrane protein that can be modified to carry out more complex functions going forward. With this, and the other work seen in this review, protein designs can begin to target membrane bound functions that seemed unobtainable previously.

In 2020 Ma et al. designed a proton conducting transmembrane protein that exceeded previous proteinaceous systems [55]. The group began with substituting glutamic acid (Glu or E) residues in the X site of the elastin-like repeat GVGXG_n_. Three variants with different charge densities named E72, HC_E35 and DC_E108 were expressed for testing. The membrane showed a proton conductivity of 18.5 mS/cm at a relative humidity (RH) of 90%. These authors believe their design can be used for the creation of implantable devices.

Xu, Baker, and others used protein design to generate a transmembrane pore that could selectively transport K ions and screen out Na or Ca [56]. They used Rosetta to generate a double-layered barrel of alpha helices that contains a hydrophobic exterior and a hydrophilic interior with a specified pore size based on the number of helices. The inner cavity was lined with polar residues that allowed ions to pass through based on their size and could be inhibited by common Na channel blockers. The authors later expanded the cavity size and allow for the transport of the larger molecule Alexa Fluor 488. Two transmembrane proteins showed a selectivity based on their pore size. Their work shows the potential of computational design to expand more than simple soluble proteins and enzymes and paves the way for studies that can fine tune the pore size on a membrane and see how cell physiology is changed in response.

## 4. Design of DNA/RNA Binding Proteins

A significant portion of the human proteome encodes for proteins that can bind to nucleic acids. DNA binding proteins (DBP) are responsible for replication, initiation and regulation of transcription, organization, compaction, and modification of DNA [57]. RNA binding proteins (RBP) control post-transcriptional processes like mRNA transport, modulation of translation, splicing and ultimately decay [58]. Applying de novo design to create proteins that bind to nucleic acids will allow to us to further our understanding of what drives binding between the two, potentially leading to novel therapeutics, genetic editing tools, and the ability to repress or enhance genes.

In 2020 Inamoto et al. combined rational design with phage-assisted continuous evolution (PACE) to create MEF, a protein that selectively binds to the E-box motif (enhancer box, CACGTG) where the transcription factor Myc binds [59]. Unregulated Myc is associated with 50% of cancers. The researchers used their previously designed ME47, a hybrid of the Max basic region and the E47 helix loop helix (HLH) as their starting point. ME47 was proven effective in inhibiting tumor growth in mouse models of breast cancer but tended to misfold so additional modifications to increase stability were made. First PACE was used to find non rational modifications that increased stability. Next mutations in ME47′s HLH were made to eliminate disulfide formation and a FosW leucine zipper was fused to compensate for the mutation. This resulted in MEF having a three-fold greater binding to E-box and four-fold increased specificity for E-box over nonspecific DNA.

Lebar and Jerala have had success with designing peptides that can act as transcription activators [60]. They developed a tunable set of heterodimeric peptides that form coiled coils and based on the peptide sequence, have an array of different functions, from cellular localization to transcriptional activation. These peptides were used to construct a CRISPR-Cas9 transcriptional activator, which increased the cellular response to certain light and chemical stimuli. Importantly, this was all done in mammalian cells, showing the impact protein design can have on medical fields. Their work sets up protein design to tackle a wide array of biological functions and take part in synthetic biology through regulation of cellular processes.

Although the field of de novo designed proteins capable of binding to nucleic acids is relatively under researched, there have been advances that use other design methods which may offer engineering insights. Walker and Varani discuss their approach in designing peptides capable of binding to RNA, which focused mainly on structure based peptidomimetics [61]. These are designed peptides meant to mimic the sequence and structure of proteins known to interact with RNA. This method uses stable well-known secondary structures like β-hairpins and α-helices to provide a backbone structure that, when combined with energetically favorable sidechains, can lead to greater binding affinity. The authors state “β-hairpins are of particular interest for targeting RNA as many RNA-binding proteins exploit β-sheet structures” and chose to use a macrocyclic β-hairpin in their designs. A stable β-hairpin can be created with two anti-parallel β-strands stabilized by two β-hairpin turn inducers. The authors also state that “this class of cyclic β-hairpins also has the size and shape to match major groove RNA”. The group then goes on to provide examples of peptidomimetics which target the BIV-TAR-Tat interaction, HIV-TAR and Rev-RRE (Rev Response Element) interaction in HIV and pre-mircoRNA-21.

Protein-protein interactions (PPIs) have also been used to extend genetic functions in addition to enzymatic ones. Smith, Savery et al. used PPIs to develop an artificial transcription factor, fusing an RNA polymerase recruitment peptide to a separate DNA binding peptide [62]. Both the RNA pol and the DNA binding section had one half of a PPI interface attached, and in vivo the two pieces would come together to develop a larger assembly. This work shows how PPIs can be used to control larger genetic circuits and paves the way for protein design to be used in synthetic biology and genetics for greater control over genetic pathways.

**Figure 3:**
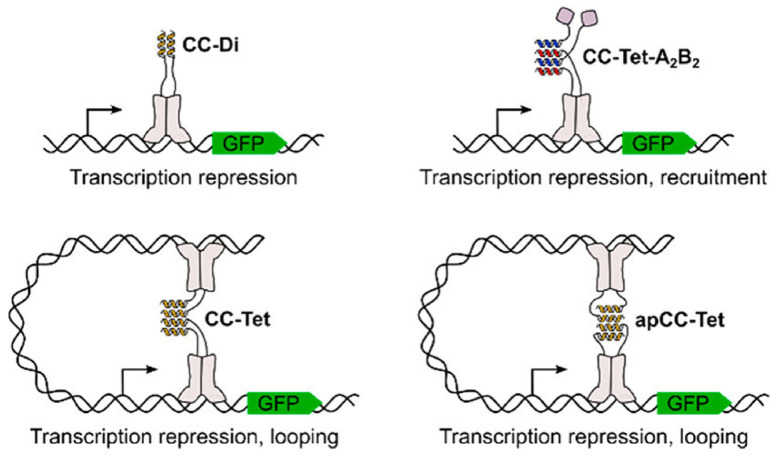
Using protein-protein interactions to rescue the function of a DNA binding protein. Edgell and coauthors attached their associating helices to the segments of the LacI repressor. Depending on the sequence of the de novo helical pairs the authors could form either two or four helix bundles, which provided them a method of tuning the function of their engineered repressor protein. Reprinted with permission from C. Edgell, L., Smith, A.J., Beesley, J.L., Savery, N.J. and Woolfson, D.N. *De Novo* Designed Protein-Interaction Modules for In-Cell Applications. *ACS Synth Biol*. Volume 9, no. 2, pp. 427–436, February 2020.

Edgell and her coauthors have also used de novo designed PPIs to promote formation of higher order structures that bind DNA [63]. Their work took a helix that can spontaneously form into a coiled coil dimer in solution and attached it to the natural repressor protein LacI, which was modified to have its oligomerization domain removed. They rescued some of the dimerization and DNA binding capabilities of the repressor protein (Figure 3). These efforts were improved when the authors modified their de novo designs to increase the oligomerization state from two helices to four (Figure 3). The addition of two more LacI subunits reproduced its wildtype activity, where it represses DNA as a tetramer. This activity could also be modified by changing the orientation of the helices relative to one another. In this sense, the group has developed a scalable set of oligomers where one can take any of the designs and use them to join together proteins in a spatiotemporal manner. These assemblies are effective in vivo and can lead to new therapies that regulate transcription from the sum of many collective inputs.

## 5. Packing of the Hydrophobic Core and Structural Stability

PPIs are growing more complex. A deeper understanding of the advances in core packing and the increased structural stability must also be looked into. This work is included here as the advances in the core structure stability will lead to proteins that can adopt more complex shapes, aided by the stability of tighter cores. We will go through a few illustrative examples that have been recently published.

In 2019 Catrina Nguyen et al. investigated the contributions of a fully hydrophobic core and hydrophilic surface to a protein’s thermostability [64]. This was done by creating two hybrid chimera proteins which combined the buried fully hydrophobic core residues and polar surface residues of UVF with the surface and core residues of EnHD (hybrid one had UVF’s core and EnHD’s surface and the second hybrid was vice versa). UVF is a de novo designed protein based on the backbone structure of the naturally occurring drosophila transcription factor EnHD. UVF has been proven to be remarkably thermostable whereas EnHD is not. The researchers performed molecular dynamic simulations of both proteins (UVF, EnHD) at different temperature ranges, and two others. They also performed coarse-grained simulations and calculated specific heat, enthalpy, entropy and free energy using weighted-histogram analysis method. They found that both a hydrophobic core and hydrophilic surface both increase thermostability. The authors propose that UVF’s hydrophobic core is responsible for entropic stabilization whereas its hydrophilic surface provides enthalpic stabilization.

It is understood that hydrophobic core packing plays a significant role in the thermostability of a protein [65]. It is also understood that de novo designed proteins tend to be more thermostable, making them a useful tool to study how residue changes can affect overall stability. Rie Koga et al. conducted an experiment where they tested how thermostability changed after replacing larger hydrophobic core residues like Leu and Ile with smaller ones like Val in a de novo designed protein. Different mutations were made ranging from 1 residue changes to 10 residue changes. They found that even after substituting in 10 valine residues and effectively creating a mostly valine core, (30 out of 34 core residues) the de novo protein remained highly thermostable. The researchers also looked at the effect of altering local backbone structures by adding one residue to two residue loops and removing one residue from three residue loops. These changes led to the protein losing its ability to fold correctly, however the authors state, “this does not imply de novo designed structures are vulnerable to any loop changes” and that more loop types should be tested. The authors also state that many studies attempt to increase protein thermostability for industrial applications by altering side chains but remodeling backbone structure with ideal ones could be an alternative way of increasing stability.

In 2020 Banach et al. tested 4 de novo designed proteins each differing in single mutations with the goal of understanding how a single residue change can lead to complete reorganization of a monomeric 3 α ɑ and 4β + ɑ folds [66]. The designed proteins are 56 amino acids long but were able to represent diverse 3D structures after a single mutation. The authors used 2 models to characterize the folding differences between the 4 proteins. First, the early-stage model was applied to determine structural differences not disclosed in secondary-structure classification. Next, the Fuzzy Oil Drop model was used to determine residues that are part of the hydrophobic core and residues located on the surface. The authors concluded that protein folding is driven by a specific synergy where the development of a micelle type structure can happen in diverse ways. The authors state that their findings are closely related to the amyloidogenesis process.

WA20, a de novo designed 4-helix bundle dimer was recently used to make protein nanobuilding blocks which create self-assembling polyhedral and chain like complexes. In 2020 Kimura et al. made design improvements to WA20 by mutating residues that would lead to an increased stability of both the hydrophobic core and helices. These efforts led to the creation of the highly thermostable protein Super WA20. (SUWA) [67]. Compared to the midpoint of WA20 (T_m_ = 75 °C), Super WA20 (T_m_ = 122 °C) displays a much higher midpoint. The authors state that stable nanoscale pillars of protein nanobuilding blocks and be used to create new types of self-assembling nanostructures.

A key aspect of protein design is the robustness of the scaffolds. To make minute changes and isolate their effect on function the protein must be able to withstand a series of mutations without misfolding. This is just as important for PPIs, and addressed by Edgell, Woolfson and colleagues [68]. Their work developing a series of homotetrameric coiled coils identifies key residues and positions that contribute to stability of these motifs, and recommends certain sequences that are more robust than others for future work. The authors note that incorporation of a Gln residue improves stability more than salt bridges of Glu and Lys. The avoidance of a salt bridge, but an increase in stability may lead to stronger designs in the future. Though the authors did not focus on the core packing itself, their work contributes to the overall stability in a significant way.

PPIs are also being extended past simple dimer interfaces. Chen, DiMaio, and their coauthors developed a PPI set that extends to whole 2-D arrays [69]. Starting with a helical bundle, the surface was redesigned in Rosetta to accommodate interactions that provide specific contacts allowing for ordered protein lattices. Importantly, the authors were able to apply this technique to develop different protein lattice topologies. Their work led to programmable 2-D structures made of repeating units. This work can begin to bridge protein design with biomaterials in a notable way, converting functional enzymatic alpha helical bundle proteins into functional enzymatic protein materials.

## 6. Design of Protein Interfaces

### 6.1. Protein-Protein Interactions

Protein-Protein interactions (PPIs) in nature are immensely important, driving a whole host of chemical reactions and other cellular processes [70]. The field of de novo protein design has long sought to identify the minimal requirements that drive this process and better understand how this is carried out in nature and repurpose it for other functions. Early studies of PPIs have been able to form rudimentary examples using metal ions to tether together subunits. Since then, there have been many advances in both the complexity of the binding sites and the level of detail of the orientation. We will go through some recent examples of them here.

**Figure 4:**
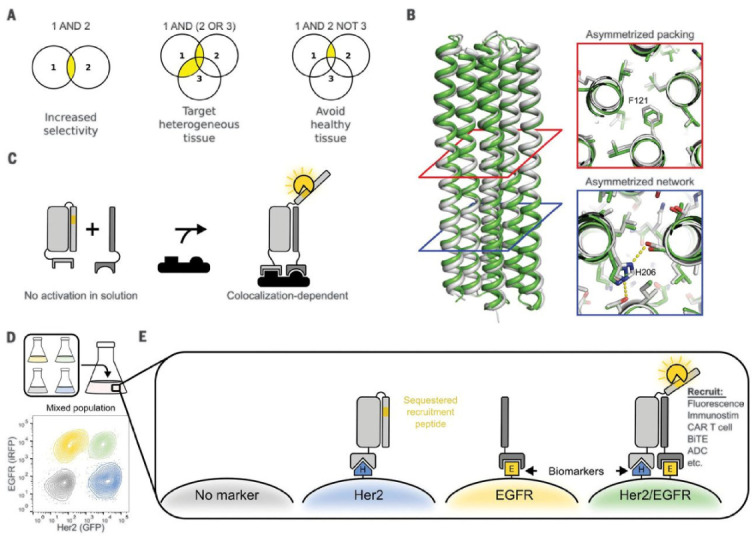
Incorporation of protein logic with the LOCKR system of proteins. (**A**) Combinations of protein-protien interactions that lead to different logic functions. (**B**) Structure of the scaffold “cage” protein used to create the Co-LOCKR system. (**C**) Colocalization of proteins is required for detection. Proteins are designed to not interact significantly in solution, but only when they are colocalized. (**D**) Flow cytometry can discriminate cells based on their surface antigens due to the Co-LOCKR system. (**E**) Depiction of effector protein recruitment based on surface antigens. Figure subunits F and G removed for clarity. Reprinted with permission from Lajoie, M.J. et al. Designed protein logic to target cells with precise combinations of surface antigens. *Science* Volume 369, no. 6511, pp. 1637–1643, September 2020.

In 2019 Robert Langan et al. designed LOCKR a tunable and generalizable protein switch [71]. In this system a key is added in trans to the LOCKR switchable system and activates protein function. The group used three examples to display the generality of the LOCKR system which included protein degradation mediated by the cODC degron, pro-apoptotic peptide Bim binding to Bcl2, and protein localization via a nuclear export sequence. Lajoie et al. recently took this milestone and expanded it by redesigning LOCKR to create a colocalization-dependent LOCKR (Co-LOCKR) [72]. Co-LOCKR is a protein capable of performing AND, OR and NOT boolean operations where conformational changes only happen after all conditions are met (Figure 4). If the target cell only presents one antigen, only the key or the cage will bind but if two antigens are present both will bind. Co-LOCKR represents a breakthrough for biological CAR-T strategies because CAR does not need to be redesigned to be specific to the antigen of a different tumor [73].

Further advancements related to de novo designed protein logic gates were made in October of 2020 when Chen et al. described the design of a 2-input AND, OR, NAND, NOR, XNOR, and NOT logic gates and investigated 3-input OR, AND and disjunctive normal form gates [74]. The researchers were able to test the efficacy of these gates with arbitrary protein units like split enzymes and transcriptional machinery in vitro, in yeast and in primary human T cells. The gates were used in T cells to regulate the expression of TIM3, a gene associated with T cell exhaustion.

Achieving binding reactions are often the first step toward larger functions. In some cases, those are other signaling events, like the LOCKR system, but in nature binding can also be used to signal the need for metabolic activity. Glasgow, Kortemme et al. have taken these reactions to the next step. They created a protein that undergoes enzymatic activity only when it binds a signaling molecule [75]. To achieve this result the authors first decided on a signal, choosing farnesyl pyrophosphate (FPP), a common metabolite. They designed a dimer interface that, together, bound FPP, and was fused to half of a reporter enzyme murine dihydrofolate reductase, which is required for cellular metabolism and therefore survival. This process allowed the authors to test the efficacy of their system by adding both FPP and inhibiting the natural dihydrofolate reductase. This work begins to bring functional responses to the binding field. Through their work the authors rewired a biological pathway, necessitating the presence of FPP for cell survival. Going forward, this tactic of combining small molecule binding with PPIs can be applied to a variety of other metabolites and enzymatic reactions.

Linking binding reactions to other signaling events is a significant step. This has been shown with the LOCKR system, using other proteins, but the work of Schnatz, Koder and their colleagues apply this to small molecules [76]. Taking a cue from biologically common intrinsically disordered proteins, the authors supercharged the sequence of a de novo designed protein named H4 and were able to show that it was unstable in weakly ionic conditions but regained its structure upon addition of salts or spermine—A polycationic molecule. Interestingly, this behavior was not limited to one de novo protein, but could be engineered into natural proteins as well. They increased the surface charge of cytochrome *b*_562_ and subjected it to the same experiments observing similar behavior. They conclude that this treatment of proteins can be used to add a form of allostery to any protein by using surface charge to disrupt the folding, and but retrieving function by balancing the charges to rescue the structure.

### 6.2. Antimicrobial Peptides and Other Therapeutics

Antimicrobial peptides (AMPs) are short peptides that either disrupt biofilm formation or kill the bacteria off entirely. Though not typically included in discussions of protein design we believe they represent valuable examples of using structural data from proteins to create binding interfaces and engineer rudimentary PPIs.

Chevalier et al. published a study where they designed and tested 22,660 de novo mini protein binders and 6,286 control sequences [77]. The mini proteins were 37–43 residues in length, contained multiple hydrophobic residues and were designed to bind influenza haemagglutinin and botulinum neurotoxin B. The control mini proteins were used to learn about binding and folding. The study identified 2,618 high affinity binders that are highly stable and do not lose activity in high temperatures.

Wang et al. designed three short α-helix containing antimicrobial peptides called GH8, GH12 and GH16 [78]. The sequences of the three peptides were GH8, GLLWHLLH-NH_2_; GH12, GLLWHLLHHLLH-NH_2_; and GH16, GLLWHLLHHLLHH-NH_2_. Minimal inhibitory concentration (MIC) and minimal bactericidal concentration (MBC) were found for all three peptides to 8 cariogenic bacterial strains and one monospecies static biofilm. Of the three peptides tested GH12 had the lowest MIC and MBC at a MIC of 4.0–8.0 µg/mL and MBC of 8.0–32.0 µg/mL making it the most promising candidate of group. In vitro GH12 was shown to have antimicrobial activity against cariogenic bacteria and biofilms along with “little toxic effect” on the viability of human gingival fibroblasts.

In a study published in 2019 by Charles Chen et al. detailed a new simulation-guided rational design approach that can be used for designing de novo AMPs [79]. The team used folding-partitioning molecular dynamic simulations to predict structures and improve their functional properties. The group began with a polyleucine peptide, then added a charged lysine and glycine on each terminus to improve solubility. Using a 14 residue template they were able to design a AMP with only 4 different types of amino acids (LDKA) that was able to form pores in common Gram-positive and Gram-negative microbial membranes. Hemolysis assays were used and determined that the de novo designed AMP showed “negligible” damage to red blood cells. This study also demonstrates that molecular dynamics simulations can predict AMP structures and fine-tune their functional properties for the development of novel therapeutic peptides.

Boris Vishnepolsky et al. developed a tool for AMP prediction called the special prediction (SP) tool [80]. An algorithm for designing de novo AMPs based off SP called DSP was created. The group used their algorithm to create AMPs capable of attacking Gram-negative bacteria. The AMPs were tested in vitro against E. Coli ATCC 25922, 14 out of 15 of the peptides performed as predicted. Improvements were made on the peptides like synthesizing the D-enantiomers of the AMPs which ultimately led to improved stability against protease digestion. This led to the creation of the peptides SP15D which has the lowest minimum inhibitory concentration compared to all peptides in the DBAASP database and SP4. Both SP15 and SP4 change membrane morphology and but at concentrations near their MIC the peptides behave differently. SP4 affects membrane structure and SP15 does not. The designed AMPs showed no hemolytic or cytotoxic effects. With antibiotic resistance continuing to be a threat [81], the virtually unlimited possibilities de novo designed AMPs offer us another avenue to continue the fight against continuously evolving pathogens.

### 6.3. Using Protein Design to Combat COVID-19

More recently the study of PPIs has been used to develop interventions to the SARS-CoV-2 virus (commonly known as COVID-19), the cause of the worldwide pandemic [82]. Various groups have been using de novo designed peptides to both study the function of this virus as well as develop peptides that can interfere with the attachment of the spike protein to angiotensin 2 [83]. This important work not only shows how protein design can be used to understand viral protein function, but is a valuable demonstration of the applications of this technique to solving real world problems.

Linsky and coauthors used a varied approach to create an effective decoy of the Angiotensin converting enzyme (ACE2) seen on the cell surface [84]. Using structures of the COVID spike protein and ACE2 they reproduced this binding interface computationally, and refined those interactions with a combination of directed evolution and de novo design. Their final protein can occupy all three sites on the spike proteins receptor binding domain (RBD). This protein was also engineered to be robust and thermostable which allowed them to test it as a nasally delivered therapy to the virus. These trials showed 100% survival for animals introduced to the virus compared to the control group where all animals became compromised and required euthanasia. Their work is significant for more than developing a treatment for this pandemic. They also established a pipeline to effectively develop and administer mimics to other disease proteins.

Larue and Sharma take a similar approach to combating this virus [85]. They, along with their collaborators, designed a panel of peptide mimics that form alpha helices and reproduce key interactions between the SARS-CoV-2 spike protein and ACE2. Their peptides were developed by modeling interactions from a related coronavirus, and that has enabled them to block not only the SARS-CoV-2 virus, but also common cold coronaviruses, highlighting how PPIs can be easily expanded to tackle other diseases.

Cao and colleagues have taken a similar approach of targeting the ACE2 binding interface with the COVID spike protein’s RBD [86]. In this case however they used Rosetta to design a specific scaffold that matches interactions from the RBD to a scaffold. In addition to the interactions made to the RBD, Rosetta was able to make additional contacts to the spike protein that increased the affinity, making it a more effective binding partner in nature. However, in addition to this strategy, this team also investigated making a library of high affinity mini-binders that could target many different sections of the COVID spike protein RBD, thereby inhibiting it at more than one site. These minibinders were incredibly effective with IC50′s in the pM-nM range, and provide a new way to develop therapeutics aside from the common antibody technologies commonly thought of. They are a fraction of the size of standard antibodies and can attack the virus from many different aspects of the spike protein.

### 6.4. Using De Novo Designed PPIs to Fight Cancer

Protein design has an active role to play in the fight against cancer by inhibiting the pathways that promote tumor metastasis or other metabolic functions. Below are some recent examples of these studies.

Ibara and Bartlett recently used a series of computational programs to perform alanine scanning experiments on a designed helix that binds to MCL-1, a membrane spanning protein often seen in cancer cells [87,88]. Alanine scanning is the systematic replacement of amino acids with alanine to measure the effect of their interaction contributes to the overall binding affinity, and the authors validated a series of computationally predicted residues that trigger protein activation. Their work brings protein design further into the cancer battle and will help identify residues that are integral to PPIs and regulate cancer. Using natural proteins would have not shown as clear a relationship due to evolutionary complications, making this the ideal system to develop therapeutics.

Johannes and Hird also sought to inhibit MCL-1 function through protein design [89]. However, compared to the previous work of Bartlett, they designed a small peptide that would interfere with this protein function rather than investigate alanine scanning options. Their small peptide was designed to match with key residues on the MCL surface and shut down the protein function. Their work is important toward understanding this cancer-linked protein as the peptide design led to a deeper understanding of the roles of MCL-1 function.

Further work from Fletcher and collages details their efforts to build stronger interfaces to disrupt naturally occurring MCL interactions, both as a cancer therapy and a tool to better understand PPIs [90]. This work used alanine scanning to identify key structural residues of the MCL-NOXA-B PPI. These results guided the development of a peptide that can disrupt these assemblies in vitro by binding to the MCL-1 protein preferentially. Interestingly, the authors designed their peptide such that it requires MCL-1 to be effective. Its hydrophobic patterning and surface charge must be effectively matched by the MCL-1 protein to promote folding into an alpha helix. This valuable methodology will be key to future designs aimed at developing allosteric effects from de novo proteins or other triggers based on the environment. The authors also highlight how their work establishes de novo designed peptides as effective and selective tools for controlling PPIs in biology.

In addition to blocking MCL-1 interactions to fight cancer, PPIs have been used to target p53, a protein implicated in approximately 50% of gene mutations identified in tumor cells. Kamagata and coauthors developed a novel way to design proteins that bind to intrinsically disordered regions (IDRs)-commonly occurring pieces of proteins that lack a defined secondary and tertiary structure [91]. Their algorithm avoided direct modeling by eschewing the need for structures because they are targeting an IDR. They used only sequence data to design a small peptide (referred to as DP6 in their work) which binds to the p53 IDR and blocks it from binding and scanning DNA. This work is important to the PPI field as their algorithm does not rely on any structural data, but simply matches amino acids based on how much energy is given off and the primary sequence. The lack of a predefined tertiary structure is key, as this process does not design a peptide with a specific confirmation. With this work groups can target a variety of proteins that require extra pieces to fold (metal ions, chaperon proteins, etc.) for interactions, all without using a supercomputer to direct these studies; impressively, this was done on a desktop computer.

Relatedly, Liu, Xing et al. designed a series of small peptide inhibitors to block epidermal growth factor receptor proteins from dimerizing [92]. Similar to the work of Hird, and Fletcher this small protein was designed to target key residues at the dimer interface and act as a therapeutic to lung cancer, and importantly have a high efficacy toward both the wildtype EGFR protein, and certain drug resistant mutant strains. This work lends further support to the idea that protein design is a valid cancer fighting technique, and can support current treatments like Gefitnib and Erloitnib, common small molecule drugs used to target lung cancer.

Interleukins are a class of glycoproteins capable of regulating immune response. Designing proteins that are capable of binding to interleukin binding sites will potentially lead to therapeutic candidates. In 2019 Silva et al. designed Neoleukin-2/15 a interleukin-2 (IL-2) and interleukin-15 (IL-15) mimic [93]. Neoleukin-2/15 shares only its binding site with IL-2 and IL-15 but otherwise has a different amino acid sequence and topology. The de novo designed mimic was tested and showed increased therapeutic activity compared to IL-2 in mouse models of melanoma and colon cancer.

In 2019 Grisoni et al. used machine learning to generate 1000 de novo designed anticancer peptides [94]. From those 1000 designs, 14 were expressed and tested in vitro on lung cancer (A549) and breast cancer (MCF7) cell lines, with 5 showing anticancer effects. Incorporating machine learning into peptide/protein design has been proven to be useful in developing novel therapeutics.

Published in 2019, Junfeng Shi and Joel Schneider de novo designed a peptide, DVP-1P that is unfolded until it interacts with alkaline phosphatase (ALP), an enzyme that is overexpressed on the surface of some cells including cancer cells [95]. When the peptide interacts with ALP it causes a conformational shift leading to “cell-surface-induced folding” which can perturb and, in high concentration, lyse the cell membrane. The authors state that peptides activity correlates with how much enzyme is expressed on the surface of the cell meaning there is a basis for functional control.

### 6.5. Methods of Identifying PPIs

Many of the PPIs we discuss are in vivo or designed to be active within a live cell. Monitoring sucess in these environments is difficult due to the dense nature and crowding of biological systems. It is important to develop new methodologies to view and validate these interactions alongside the development of PPIs themselves. Although we have gone through many methods in the papers above, we wanted to highlight a small set of new techniques to monitor PPIs.

Zhao and Dmchowski have developed a new way of identifying PPIs in vivo with Xe NMR spectroscopy [96]. In this method the authors treat a foreign protein with Xe compounds and feed it to cells. Where they detect this signal can tell them what protein is contacting in vivo and make more careful determinations of which amino acids are involved. This technique will help develop de novo proteins as cellular tools by allowing scientists to follow the path of their designed protein in natural systems ensuring it is meeting its target and acting as expected.

Yudenko Gushchin and coworkers have also developed a similar assay that will help probe PPIs in nature, and importantly in anaerobic situations [97]. This assay utilizes a flavin-based fluorescent protein (FbFP) that is cut in half, and each half attached to a piece of the PPI in question. If the proteins for a strong dimer then fluorescence is recovered in vivo, and with a high signal that is clear to detect. Importantly, this assay can be used in both *E. coli* and human cells, which will help groups better detect a variety of PPIs in wildly different systems. They are also maximally functional in anaerobic conditions allowing for probing of PPIs in new environments.

## 7. New Algorithms for Protein Design

Alongside the many advances in functions and binding and packing interactions the complexity of designs themselves have grown. Improved algorithms are needed to calculate the lowest energy conformations and better determine the ideal folding and interactions. In this section we will go through some recent developments in computational programs, and their applications. For a more comprehensive analysis and perspective on computational protein design we recommend the following articles [98,99].

The Baker lab has taken a general approach to small molecule binding, creating an algorithm that takes advantage of the structural stability, and diversity, of a naturally occurring protein family, NTF-2. Basanta and coauthors developed an enumerative algorithm that first docks a ligand into a protein cavity, then systematically samples a variety of different amino acid combinations in that cavity to find the most ideal binding partner [100]. Overall they were able to sample over 1600 different protein variations, able to bind different ligands. Importantly, this algorithm was able to screen out variants that are inherently unstable and could misfold or cause other issues. This work is significant as it starts with a stably folded scaffold, of which there are many known motifs, and generates a library of proteins, which can greatly expand the reach of proteins designed by exhaustively searching many variations and matching them to specific ligands. Groups can eschew searching the PDB for an idealized cavity that meets the biophysical needs of a specific ligand and instead follow the enumerative algorithm developed here.

Strokach, Kim, and colleagues have also taken an interesting algorithmic approach to protein design [101]. Their program, ProteinSolver, utilizes a deep graph neural network to design proteins incorporating the calculated stability at each site as a measure of efficacy. This program was trained on over 70 million sequences, and 80-thousand solved structures to develop a library of proteins that are analyzed for stability before being ranked. The authors validated ProteinSolver by reproducing the structure of human serum albumin and characterize the “design” in vitro to prove the accuracy. This freely available program will be useful for the next generation of designs by incorporating a new way of analyzing existing structures in its neural net.

Skalic De Fabritiis and coworkers have also developed an algorithm that can better design binding pockets for small molecules [102]. However, this algorithm uses machine learning approaches to sculpt a binding site based on the shape, and then chemical properties. With these pieces of information their program comes up with SMILES tokens, which can be used to rebuild the protein in many different computer programs. In addition to better building proteins, the SMILES tokens can also be used for evolutionary analyses by providing a number of comparative examples for small molecule binding sites.

Lucas and Kortemme have developed a new algorithm that combines PDB screened ligand binding sites with the established Rosetta design programs to create a library of ligand binding proteins that can bind arbitrary small molecules [103]. They were able to identify short peptide segments that form common contact pairs to small molecules and are highly represented in the PDB. Taking these repeating patterns, they used Rosetta software to graft these segments into new protein structures and form ligand binding proteins for a large set of ligands. This work is significant to the field of small molecule design as it provides a new method to develop a large number of small molecule binding proteins. This work is also able to identify small molecule binding sites not previously known in natural proteins through similar means of identifying repeating binding patterns in the PDB. This will be a notable use of protein design to bioinformatics providing a new way of identifying ligand interactions.

In May 2020 Sesterhenn et al. created a new design algorithm called Topobuilder meant for creating scaffolds for irregular and discontinuous neutralization epitopes [104]. They proved the effectiveness of their algorithm by designing immunogens that mimic epitopes for respiratory syncytial virus (RSV). Their designs were tested in vitro where they were found to bind with high affinity to site specific RSV neutralizing antibodies and in vivo where they increased antibody count and quality of response compared to other RSV boosters. The authors state that their strategy can be applied to creating functional sites with high complexity.

In 2020 Liu et al. investigated if protein structure prediction could be improved by using loop-specific sampling strategy [105]. The strategy consisted of two stages, first a global exploration phase where the conformational space was explored and produced topologies similar to the native protein. Next in the loop perturbation phase meant to increase the accuracy of the conformation via a differential evolution algorithm. This model obtained a template modeling score of ≥0.5 on 95 standard test proteins. These findings can be applied to better assess how secondary structures are connected improving the ability to predict structures.

## 8. Design from the Perspective of the Binding Pair

We have been showing examples exclusively of proteins being designed to bind to a specific small molecule, nucleic acid, or other protein. However, for context we thought we would highlight a couple of articles that show the opposite approach, matching the ligand back to the protein itself.

Interestingly, the search for ligand binding proteins does not have to be led from the protein-side, rather the molecule can be designed from the protein structure. Leal and coworkers did that in a recent paper, targeting the envelop protein of dengue fever [106]. Through a lead optimization process the authors prepared a series of substituted pyrimidines that could fit into the pocket of the coat protein and stop it from undergoing a conformational change that allows the viral DNA to get into the host cell. These molecules showed a range of activity with the four most effective ones limiting viral activity at a concentration of 1 uM, and the top two most effective compounds working against multiple serotypes of the virus. This work, though not protein design as we have been discussing, provides an interesting alternative to describing the interactions at the binding site. Through these analyses, groups are able to identify the main contributing factors. This work does not help describe protein function in the same way as other projects discussed here, but provides valuable support to these efforts.

Similar to the work of Leal and coworkers, the approach of starting with the binding partner can be used in other ways. Liu and coworkers used this bottom up approach to develop a library of DNA binding sites to generate genetic promoters with variable affinities [107]. By placing the binding site of an allosteric transcription factor at different points between a −35 and −10 sites of an inducible promoter, and by tuning the sequence of the DNA, they were able to tune the strength and inducible nature of the promoter region. The authors were able to tune the strength of this region effectively and show a large functional range. The use of allosteric transcription factors opens this work up to a whole range of biosensor and small molecule detection assays. Combined with the approaches to small molecule design seen elsewhere in this paper one can conceive the design of a transcription factor de novo that can interface with a piece of DNA, also made de novo, to foster a wholly synthetic genetic system.

## 9. Conclusions

There have been many impressive recent advances in de novo design, and the field continues to better understand the mechanisms underlying protein folding and binding of small molecules, DNA, or other proteins. All this work will help biochemists reach the ultimate goal of predicting structure and function of de novo proteins from an amino acid sequence, and generate specific binding interactions for any ligand or binding partner. Protein design has made impressive steps in this regard bringing new logic-functions to proteins [72], and developing new and efficient ways of designing small molecule binding sites [40]. There has also been a notable increase in the number of algorithms that are being developed to assist these efforts, supported by computational power, machine learning capabilities, and more structures in the PDB to learn from. Coupled with the new advances in deep-learning protein structure prediction, such as those recently announced from Google [108], the future of this field is extremely promising.

## References

[1] Vuignier K., Schappler J., Veuthey J.-L., Carrupt P.-A., Martel S. (2010). Drug–protein binding: A critical review of analytical tools. Anal. Bioanal. Chem..

[2] Siggers T., Gordân R. (2014). Protein–DNA binding: Complexities and multi-protein codes. Nucleic Acids Res..

[3] Feng Y., Walsh C.A. (2001). Protein–Protein interactions, cytoskeletal regulation and neuronal migration. Nat. Rev. Neurosci..

[4] Dutton P.L., Moser C.C. (2011). Engineering enzymes. Faraday Discuss..

[5] Lichtenstein B.R., Farid T.A., Kodali G., Solomon L.A., Anderson J.R., Sheehan M.M., Ennist N.M., Fry B.A., Chobot S.E., Bialas C. (2012). Engineering oxidoreductases: Maquette proteins designed from scratch. Biochem. Soc. Trans..

[6] Macdonald J.R., Johnson W.C. (2001). Environmental features are important in determining protein secondary structure. Protein Sci..

[7] Huang L.-S., Cobessi D., Tung E.Y., Berry E.A. (2005). Binding of the Respiratory Chain Inhibitor Antimycin to the Mitochondrial bc1 Complex: A New Crystal Structure Reveals an Altered Intramolecular Hydrogen-bonding Pattern. J. Mol. Biol..

[8] Ho S.P., DeGrado W.F. (1987). Design of a 4-helix bundle protein: Synthesis of peptides which self-associate into a helical protein. J. Am. Chem. Soc..

[9] Regan L., DeGrado W.F., Landegren U., Kaiser R., Caskey C., Hood L. (1988). Characterization of a helical protein designed from first principles. Science.

[10] Koder R.L., Anderson J.L.R., Solomon L.A., Reddy K.S., Moser C.C., Dutton P.L. (2009). Design and engineering of an O_2_ transport protein. Nat. Cell Biol..

[11] Jiang L., Althoff E.A., Clemente F.R., Doyle L., Röthlisberger D., Zanghellini A., Gallaher J.L., Betker J.L., Tanaka F., Barbas C.F. (2008). De Novo Computational Design of Retro-Aldol Enzymes. Science.

[12] Anderson J.L.R., Armstrong C.T., Kodali G., Lichtenstein B.R., Watkins D.W., Mancini J.A., Boyle A.L., Farid T.A., Crump M.P., Moser C.C. (2014). Constructing a man-made c-type cytochrome maquette in vivo: Electron transfer, oxygen transport and conversion to a photoactive light harvesting maquette. Chem. Sci..

[13] Farid A.T., Kodali G., Solomon A.L., Lichtenstein B.R., Sheehan M.M., Fry A.B., Bialas C., Ennist N.M., Siedlecki A.J., Zhao Z. (2013). Elementary tetrahelical protein design for diverse oxidoreductase functions. Nat. Chem. Biol..

[14] Currin A., Swainston N., Day P.J., Kell D.B. (2015). Synthetic biology for the directed evolution of protein biocatalysts: Navigating sequence space intelligently. Chem. Soc. Rev..

[15] Packer M.S., Liu D.R. (2015). Methods for the directed evolution of proteins. Nat. Rev. Genet..

[16] Neylon C. (2004). Chemical and biochemical strategies for the randomization of protein encoding DNA sequences: Library construction methods for directed evolution. Nucleic Acids Res..

[17] Kan S.B.J., Lewis R.D., Chen K., Arnold F.H. (2016). Directed evolution of cytochrome c for carbon–silicon bond formation: Bringing silicon to life. Science.

[18] Karanicolas J., Corn J.E., Chen I., Joachimiak L.A., Dym O., Peck S.H., Albeck S., Unger T., Hu W., Liu G. (2011). A De Novo Protein Binding Pair By Computational Design and Directed Evolution. Mol. Cell.

[19] Grayson K.J., Anderson J.L.R. (2018). Designed for life: Biocompatible de novo designed proteins and components. J. R. Soc. Interface.

[20] Huang P.-S., Boyken S.E., Baker P.-S.H.S.E.B.D. (2016). The coming of age of de novo protein design. Nat. Cell Biol..

[21] Korendovych I.V., DeGrado W.F. (2020). De novoprotein design, a retrospective. Q. Rev. Biophys..

[22] Calhoun J.R., Nastri F., Maglio O., Pavone V., Lombardi A., DeGrado W.F. (2005). Artificial diiron proteins: From structure to function. Biopolymer.

[23] Maglio O., Nastri F., De Rosales R.T.M., Faiella M., Pavone V., DeGrado W.F., Lombardi A. (2007). Diiron-containing metalloproteins: Developing functional models. Comptes Rendus Chim..

[24] Kaplan J., DeGrado W.F. (2004). De novo design of catalytic proteins. Proc. Natl. Acad. Sci. USA.

[25] Paredes A., Loh B.M., Peduzzi O.M., Reig A.J., Buettner K.M. (2020). DNA Cleavage by a De Novo Designed Protein–Titanium Complex. Inorg. Chem..

[26] Olson T.L., Espiritu E., Edwardraja S., Canarie E., Flores M., Williams J.C., Ghirlanda G., Allen J.P. (2017). Biochemical and spectroscopic characterization of dinuclear Mn-sites in artificial four-helix bundle proteins. Biochim. Biophys. Acta (BBA) Bioenerg..

[27] Chino M., Zhang S.-Q., Pirro F., Leone L., Maglio O., Lombardi A., DeGrado W.F. (2018). Spectroscopic and metal binding properties of a de novo metalloprotein binding a tetrazinc cluster. Biopolymer.

[28] Zhang S.-Q., Chino M., Liu L., Tang Y., Hu X., DeGrado W.F., Lombardi A. (2018). De Novo Design of Tetranuclear Transition Metal Clusters Stabilized by Hydrogen-Bonded Networks in Helical Bundles. J. Am. Chem. Soc..

[29] Pirro F., Schmidt N., Lincoff J., Widel Z.X., Polizzi N.F., Liu L., Therien M.J., Grabe M., Chino M., Lombardi A. (2020). Allosteric cooperation in a de novo-designed two-domain protein. Proc. Natl. Acad. Sci. USA.

[30] Mancini J.A., Pike D.H., Tyryshkin A.M., Haramaty L., Wang M.S., Poudel S., Hecht M., Nanda V. (2020). Design of a Fe 4 S 4 cluster into the core of a de novo four-helix bundle. Biotechnol. Appl. Biochem..

[31] Selvan D., Prasad P., Farquhar E.R., Shi Y., Crane S., Zhang Y., Chakraborty S. (2019). Redesign of a Copper Storage Protein into an Artificial Hydrogenase. ACS Catal..

[32] Mutter A.C., Tyryshkin A.M., Campbell I.J., Poudel S., Bennett G.N., Silberg J.J., Nanda V., Falkowski P.G. (2019). De novo design of symmetric ferredoxins that shuttle electrons in vivo. Proc. Natl. Acad. Sci. USA.

[33] Boyken S.E., Benhaim M.A., Busch F., Jia M., Bick M.J., Choi H., Klima J.C., Chen Z., Walkey C., Mileant A. (2019). De novo design of tunable, pH-driven conformational changes. Science.

[34] Robertson D.E., Farid R.S., Moser C.C., Urbauer J.L., Mulholland S.E., Pidikiti R., Lear J.D., Wand A.J., DeGrado W.F., Dutton P.L. (1994). Design and synthesis of multi-haem proteins. Nat. Cell Biol..

[35] Sykes A.G. (2000). Advances in Inorganic Chemistry: Heme-Fe Proteins.

[36] Nagarajan D., Sukumaran S., Deka G., Krishnamurthy K., Atreya H.S., Chandra N. (2018). Design of a heme-binding peptide motif adopting a β-hairpin conformation. J. Biol. Chem..

[37] Zambrano G., Nastri F., Pavone V., Lombardi A., Chino M. (2020). Use of an Artificial Miniaturized Enzyme in Hydrogen Peroxide Detection by Chemiluminescence. Sensors.

[38] Polizzi N.F., Wu Y., Lemmin T., Maxwell A.M., Zhang S.-Q., Rawson J., Beratan D.N., Therien M.J., DeGrado W.F. (2017). De novo design of a hyperstable non-natural protein–ligand complex with sub-Å accuracy. Nat. Chem..

[39] Kodali G., Mancini J.A., Solomon L.A., Episova T.V., Roach N., Hobbs C.J., Wagner P., Mass O.A., Aravindu K., Barnsley J.E. (2016). Design and engineering of water-soluble light-harvesting protein maquettes. Chem. Sci..

[40] Polizzi N.F., DeGrado W.F. (2020). A defined structural unit enables de novo design of small-molecule–binding proteins. Science.

[41] Park J., Selvaraj B., McShan A.C., Boyken S.E., Wei K.Y., Oberdorfer G., DeGrado W., Sgourakis N.G., Cuneo M.J., Myles D.A. (2019). De novo design of a homo-trimeric amantadine-binding protein. eLife.

[42] Dou J., Vorobieva A.A., Sheffler W., Doyle L.A., Park H., Bick M.J., Mao B., Foight G.W., Lee M.Y., Gagnon L.A. (2018). De novo design of a fluorescence-activating β-barrel. Nat. Cell Biol..

[43] Thomas F., Dawson W.M., Lang E.J.M., Burton A.J., Bartlett G.J., Rhys G.G., Mulholland A.J., Woolfson D.N. (2018). De Novo-Designed α-Helical Barrels as Receptors for Small Molecules. ACS Synth. Biol..

[44] Caldwell S.J., Haydon I.C., Piperidou N., Huang P.-S., Bick M.J., Sjöström H.S., Hilvert D., Baker D., Zeymer C. (2020). Tight and specific lanthanide binding in a de novo TIM barrel with a large internal cavity designed by symmetric domain fusion. Proc. Natl. Acad. Sci. USA.

[45] Karas C., Hecht M. (2020). A Strategy for Combinatorial Cavity Design in De Novo Proteins. Life.

[46] Kang S., Davidsen K., Gomez-Castillo L., Jiang H., Fu X., Li Z., Liang Y., Jahn M., Moussa M., DiMaio F. (2019). COMBINES-CID: An Efficient Method for De Novo Engineering of Highly Specific Chemically Induced Protein Dimerization Systems. J. Am. Chem. Soc..

[47] Prakash V., Ranbhor R., Ramakrishnan V. (2020). De Novo Designed Heterochiral Blue Fluorescent Protein. ACS Omega.

[48] Stenner R., Steventon J.W., Seddon A., Anderson J.L.R. (2020). A de novo peroxidase is also a promiscuous yet stereoselective carbene transferase. Proc. Natl. Acad. Sci. USA.

[49] Stenner R., Anderson J.L.R. (2020). Chemoselective N−H insertion catalyzed by a de novo carbene transferase. Biotechnol. Appl. Biochem..

[50] Gromiha M.M., Ou Y.-Y. (2013). Bioinformatics approaches for functional annotation of membrane proteins. Brief. Bioinform..

[51] Cournia Z., Allen T.W., Andricioaei I., Antonny B., Baum D., Brannigan G., Buchete N.-V., Deckman J.T., Delemotte L., Del Val C. (2015). Membrane Protein Structure, Function, and Dynamics: A Perspective from Experiments and Theory. J. Membr. Biol..

[52] Engel A., Gaub H.E. (2008). Structure and Mechanics of Membrane Proteins. Annu. Rev. Biochem..

[53] Mravic M., Thomaston J.L., Tucker M., Solomon P.E., Liu L., DeGrado W.F. (2019). Packing of apolar side chains enables accurate design of highly stable membrane proteins. Science.

[54] Curnow P., Hardy B.J., Dufour V., Arthur C.J., Stenner R., Hodgson L.R., Verkade P., Williams C., Shoemark D.K., Sessions R.B. (2020). Small-residue packing motifs modulate the structure and function of a minimal de novo membrane protein. Sci. Rep..

[55] Ma C., Dong J., Viviani M., Tulini I., Pontillo N., Maity S., Zhou Y., Roos W.H., Liu K., Herrmann A. (2020). De novo rational design of a freestanding, supercharged polypeptide, proton-conducting membrane. Sci. Adv..

[56] Xu C., Lu P., El-Din T.M.G., Pei X.Y., Johnson M.C., Uyeda A., Bick M.J., Xu Q., Jiang D., Bai H. (2020). Computational design of transmembrane pores. Nat. Cell Biol..

[57] Rohs R., Jin X., West S.M., Joshi R., Honig B., Mann R.S. (2010). Origins of Specificity in Protein-DNA Recognition. Annu. Rev. Biochem..

[58] Corley M., Burns M.C., Yeo G.W. (2020). How RNA-Binding Proteins Interact with RNA: Molecules and Mechanisms. Mol. Cell.

[59] Inamoto I., Sheoran I., Popa S.C., Hussain M., Shin J.A. (2021). Combining Rational Design and Continuous Evolution on Minimalist Proteins That Target the E-box DNA Site. ACS Chem. Biol..

[60] Lebar T., Lainšček D., Merljak E., Aupič J., Jerala R. (2020). A tunable orthogonal coiled-coil interaction toolbox for engineering mammalian cells. Nat. Chem. Biol..

[61] Walker M.J., Varani G. (2019). Design of RNA-targeting macrocyclic peptides. Methods Enzym..

[62] Smith A.J., Thomas F., Shoemark D., Woolfson D.N., Savery N.J. (2019). Guiding Biomolecular Interactions in Cells Using de Novo Protein–Protein Interfaces. ACS Synth. Biol..

[63] Edgell C.L., Smith A.J., Beesley J.L., Savery N.J., Woolfson D.N. (2020). De NovoDesigned Protein-Interaction Modules for In-Cell Applications. ACS Synth. Biol..

[64] Nguyen C., Young J.T., Slade G.G., Oliveira R.J., McCully M.E. (2019). A Dynamic Hydrophobic Core and Surface Salt Bridges Thermostabilize a Designed Three-Helix Bundle. Biophys. J..

[65] Koga R., Yamamoto M., Kosugi T., Kobayashi N., Sugiki T., Fujiwara T., Koga N. (2020). Robust folding of a de novo designed ideal protein even with most of the core mutated to valine. Proc. Natl. Acad. Sci. USA.

[66] Banach M., Fabian P., Stapor K., Konieczny L., Roterman A.I. (2020). Structure of the Hydrophobic Core Determines the 3D Protein Structure—Verification by Single Mutation Proteins. Biomolecules.

[67] Kimura N., Mochizuki K., Umezawa K., Hecht M.H., Arai R. (2019). Hyperstable De Novo Protein with a Dimeric Bisecting Topology. ACS Synth. Biol..

[68] Edgell C.L., Savery N.J., Woolfson D.N. (2020). Robust De Novo-Designed Homotetrameric Coiled Coils. Biochemistry.

[69] Chen Z., Johnson M.C., Chen J., Bick M.J., Boyken S.E., Lin B., De Yoreo J.J., Kollman J.M., Baker D., DiMaio F. (2019). Self-Assembling 2D Arrays with de Novo Protein Building Blocks. J. Am. Chem. Soc..

[70] Pagel P., Kovac S., Oesterheld M., Brauner B., Dunger-Kaltenbach I., Frishman D., Montrone C., Mark P., Stümpflen V., Mewes H.-W. (2004). The MIPS mammalian protein-protein interaction database. Bioinformatics.

[71] Langan R.A., Boyken S.E., Ng A.H., Samson J.A., Dods G., Westbrook A.M., Nguyen T.H., Lajoie M.J., Chen Z., Berger S. (2019). De novo design of bioactive protein switches. Nat. Cell Biol..

[72] Lajoie M.J., Boyken S.E., Salter A.I., Bruffey J., Rajan A., Langan R.A., Olshefsky A., Muhunthan V., Bick M.J., Gewe M. (2020). Designed protein logic to target cells with precise combinations of surface antigens. Science.

[73] Xie M., Lu P. (2020). When de novo-designed protein logics meet CAR-T therapies. Cell Res..

[74] Chen Z., Kibler R.D., Hunt A., Busch F., Pearl J., Jia M., VanAernum Z.L., Wicky B.I.M., Dods G., Liao H. (2020). De novo design of protein logic gates. Science.

[75] Glasgow A.A., Huang Y.-M., Mandell D.J., Thompson M., Ritterson R., Loshbaugh A.L., Pellegrino J., Krivacic C., Pache R.A., Barlow K.A. (2019). Computational design of a modular protein sense-response system. Science.

[76] Schnatz P.J., Brisendine J.M., Laing C.C., Everson B.H., French C.A., Molinaro P.M., Koder R.L. (2020). Designing heterotropically activated allosteric conformational switches using supercharging. Proc. Natl. Acad. Sci. USA.

[77] Chevalier A., Silva D.-A., Rocklin G.J., Hicks D.R., Vergara R., Murapa P., Bernard S.M., Zhang L., Lam K.-H., Yao G. (2017). Massively parallel de novo protein design for targeted therapeutics. Nature.

[78] Wang Y., Fan Y., Zhou Z., Tu H., Ren Q., Wang X., Ding L., Zhou X., Zhang L. (2017). De novo synthetic short antimicrobial peptides against cariogenic bacteria. Arch. Oral Biol..

[79] Chen C.H., Starr C.G., Troendle E.P., Wiedman G., Wimley W.C., Ulmschneider J.P., Ulmschneider M.B. (2019). Simulation-Guided Rational de Novo Design of a Small Pore-Forming Antimicrobial Peptide. J. Am. Chem. Soc..

[80] Vishnepolsky B., Zaalishvili G., Karapetian M., Nasrashvili T., Kuljanishvili N., Gabrielian A., Rosenthal A., Hurt D.E., Tartakovsky M., Grigolava M. (2019). De Novo Design and In Vitro Testing of Antimicrobial Peptides against Gram-Negative Bacteria. Pharmaceuticals.

[81] CDC (2019). Antibiotic Resistance Threats in the United States, 2019.

[82] Haque S.M., Ashwaq O., Sarief A., Mohamed A.K.A.J. (2020). A comprehensive review about SARS-CoV-2. Futur. Virol..

[83] Yan R., Zhang Y., Li Y., Xia L., Guo Y., Zhou Q. (2020). Structural basis for the recognition of SARS-CoV-2 by full-length human ACE2. Science.

[84] Linsky T.W., Vergara R., Codina N., Nelson J.W., Walker M.J., Su W., Barnes C.O., Hsiang T.-Y., Esser-Nobis K., Yu K. (2020). De novo design of potent and resilient hACE2 decoys to neutralize SARS-CoV-2. Science.

[85] LaRue R.C., Xing E., Kenney A.D., Zhang Y., Tuazon J.A., Li J., Yount J.S., Li P.-K., Sharma A. (2021). Rationally Designed ACE2-Derived Peptides Inhibit SARS-CoV-2. Bioconjugate Chem..

[86] Cao L., Goreshnik I., Coventry B., Case J.B., Miller L., Kozodoy L., Chen R.E., Carter L., Walls A.C., Park Y.-J. (2020). De novo design of picomolar SARS-CoV-2 miniprotein inhibitors. Science.

[87] Ibarra A.A., Bartlett G.J., Hegedüs Z., Dutt S., Hobor F., Horner K.A., Hetherington K., Spence K., Nelson A., Edwards T.A. (2019). Predicting and Experimentally Validating Hot-Spot Residues at Protein–Protein Interfaces. ACS Chem. Biol..

[88] Kelly G.L., Strasser A. (2020). Toward Targeting Antiapoptotic MCL-1 for Cancer Therapy. Annu. Rev. Cancer Biol..

[89] Johannes J.W., Bates S., Beigie C., Belmonte M.A., Breen J., Cao S., Centrella P.A., Clark M.A., Cuozzo J.W., Dumelin C.E. (2017). Structure Based Design of Non-Natural Peptidic Macrocyclic Mcl-1 Inhibitors. ACS Med. Chem. Lett..

[90] Fletcher J.M., Horner K.A., Bartlett G.J., Rhys G.G., Wilson A.J., Woolfson D.N. (2018). De novocoiled-coil peptides as scaffolds for disrupting protein–protein interactions. Chem. Sci..

[91] Kamagata K., Mano E., Itoh Y., Wakamoto T., Kitahara R., Kanbayashi S., Takahashi H., Murata A., Kameda T. (2019). Rational design using sequence information only produces a peptide that binds to the intrinsically disordered region of p53. Sci. Rep..

[92] Liu Q., Zhou J., Gao J., Ma W., Wang S., Xing L. (2020). Rational design of EGFR dimerization-disrupting peptides: A new strategy to combat drug resistance in targeted lung cancer therapy. Biochimie.

[93] Silva D.-A., Yu S., Ulge U.Y., Spangler J.B., Jude K.M., Labão-Almeida C., Ali L.R., Quijano-Rubio A., Ruterbusch M., Leung I. (2019). De novo design of potent and selective mimics of IL-2 and IL-15. Nat. Cell Biol..

[94] Grisoni F., Neuhaus C.S., Hishinuma M., Gabernet G., Hiss J.A., Kotera M., Schneider G. (2019). De novo design of anticancer peptides by ensemble artificial neural networks. J. Mol. Model..

[95] Shi J., Schneider J.P. (2019). De novo Design of Selective Membrane-Active Peptides by Enzymatic Control of Their Conformational Bias on the Cell Surface. Angew. Chem. Int. Ed..

[96] Zhao Z., Roose B.W., Zemerov S.D., Stringer M.A., Dmochowski I.J. (2020). Detecting protein–protein interactions by Xe-129 NMR. Chem. Commun..

[97] Yudenko A., Smolentseva A., Maslov I., Semenov O., Goncharov I.M., Nazarenko V.V., Maliar N.L., Borshchevskiy V., Gordeliy V., Remeeva A. (2021). Rational Design of a Split Flavin-Based Fluorescent Reporter. ACS Synth. Biol..

[98] Baker D.A. (2019). What has de novo protein design taught us about protein folding and biophysics?. Protein Sci..

[99] Kuhlman B., Bradley P. (2019). Advances in protein structure prediction and design. Nat. Rev. Mol. Cell Biol..

[100] Basanta B., Bick M.J., Bera A.K., Norn C., Chow C.M., Carter L.P., Goreshnik I., DiMaio F., Baker D. (2020). An enumerative algorithm for de novo design of proteins with diverse pocket structures. Proc. Natl. Acad. Sci. USA.

[101] Strokach A., Becerra D., Corbi-Verge C., Perez-Riba A., Kim P.M. (2020). Fast and Flexible Protein Design Using Deep Graph Neural Networks. Cell Syst..

[102] Skalic M., Jiménez J., Sabbadin D., De Fabritiis G. (2019). Shape-Based Generative Modeling for de Novo Drug Design. J. Chem. Inf. Model..

[103] Lucas J.E., Kortemme T. (2020). New computational protein design methods for de novo small molecule binding sites. PLoS Comput. Biol..

[104] Sesterhenn F., Yang C., Bonet J., Cramer J.T., Wen X., Wang Y., Chiang C.-I., Abriata L.A., Kucharska I., Castoro G. (2020). De novo protein design enables the precise induction of RSV-neutralizing antibodies. Science.

[105] Liu J., Zhou X.-G., Zhang Y., Zhang G.-J. (2019). CGLFold: A contact-assisted de novo protein structure prediction using global exploration and loop perturbation sampling algorithm. Bioinformatics.

[106] Leal E.S., Adler N.S., Fernández G.A., Gebhard L.G., Battini L., Aucar M.G., Videla M., Monge M.E., Ríos A.H.D.L., Dávila J.A.A. (2019). De novo design approaches targeting an envelope protein pocket to identify small molecules against dengue virus. Eur. J. Med. Chem..

[107] Liu X., Gupta S.T.P., Bhimsaria D., Reed J.L., A Rodríguez-Martínez J., Ansari A.Z., Raman S. (2019). De novo design of programmable inducible promoters. Nucleic Acids Res..

[108] Callaway E. (2020). ‘It will change everything’: DeepMind’s AI makes gigantic leap in solving protein structures. Nat. Cell Biol..

